# Impairment and Compensation in Dexterous Upper-Limb Function After Stroke. From the Direct Consequences of Pyramidal Tract Lesions to Behavioral Involvement of Both Upper-Limbs in Daily Activities

**DOI:** 10.3389/fnhum.2021.662006

**Published:** 2021-06-21

**Authors:** Agnès Roby-Brami, Nathanaël Jarrassé, Ross Parry

**Affiliations:** ^1^ISIR Institute of Intelligent Systems and Robotics, AGATHE Team, CNRS UMR 7222, INSERM U 1150, Sorbonne University, Paris, France; ^2^LINP2-AAPS Laboratoire Interdisciplinaire en Neurosciences, Physiologie et Psychologie: Activité Physique, Santé et Apprentissages, UPL, Paris Nanterre University, Nanterre, France

**Keywords:** rehabilitation, recovery, stroke, disability, activity, physiopathologic mechanism

## Abstract

Impairments in dexterous upper limb function are a significant cause of disability following stroke. While the physiological basis of movement deficits consequent to a lesion in the pyramidal tract is well demonstrated, specific mechanisms contributing to optimal recovery are less apparent. Various upper limb interventions (motor learning methods, neurostimulation techniques, robotics, virtual reality, and serious games) are associated with improvements in motor performance, but many patients continue to experience significant limitations with object handling in everyday activities. Exactly how we go about consolidating adaptive motor behaviors through the rehabilitation process thus remains a considerable challenge. An important part of this problem is the ability to successfully distinguish the extent to which a given gesture is determined by the neuromotor impairment and that which is determined by a compensatory mechanism. This question is particularly complicated in tasks involving manual dexterity where prehensile movements are contingent upon the task (individual digit movement, grasping, and manipulation…) and its objective (placing, two step actions…), as well as personal factors (motivation, acquired skills, and life habits…) and contextual cues related to the environment (presence of tools or assistive devices…). Presently, there remains a lack of integrative studies which differentiate processes related to structural changes associated with the neurological lesion and those related to behavioral change in response to situational constraints. In this text, we shall question the link between impairments, motor strategies and individual performance in object handling tasks. This scoping review will be based on clinical studies, and discussed in relation to more general findings about hand and upper limb function (manipulation of objects, tool use in daily life activity). We shall discuss how further quantitative studies on human manipulation in ecological contexts may provide greater insight into compensatory motor behavior in patients with a neurological impairment of dexterous upper-limb function.

## Introduction

Impairments of dexterous upper-limb function are a significant cause of disability following an acquired brain injury or stroke since they affect approximately one half of the patients in this clinical population (Jorgensen et al., 1995). Recovery of upper-limb function after stroke has been the subject of numerous studies from both fundamental and clinical perspectives. Over the two last decades, various novel therapeutic interventions have been proposed (Winstein et al., 2016a). But despite impressive preclinical advances, patient outcomes in rehabilitation often remain disappointing, “with interesting science ultimately proving difficult to translate to the clinic” (Ward and Carmichael, 2020).

Defining hand dexterity proves somewhat complicated, with different perspectives emphasized across scientific disciplines (neurophysiology, cognitive science, humanities). From an etymological point of view, dexterity concerns mainly the right dominant hand. According to a modern dictionary, dexterity is the “readiness and grace in physical activity, especially skill and ease in using the hands” (Merriam-Webster). Exquisite hand dexterity is relatively specific to the human species which possesses several anatomical attributes (e.g., large moment arms for intrinsic muscles) facilitating independent finger movements and thumb opposition for precision grips (Napier, 1961; Marzke and Marzke, 2000). Obviously, human hand dexterity is equally related to the development of human neural substrates, particularly the monosynaptic cortico-motoneuronal system (Lemon et al., 2004). Lateralization and right-hand preference is also quite specific to humans and great apes (Cochet and Byrne, 2013). In addition, the human hand is remarkably versatile, being able to adopt a great variety of postures (Napier, 1956; Kapandji, 1980; Iberall et al., 1986). The control of such a sophisticated and mobile apparatus is highly cognitive. Dexterous upper-limb function relies on skills acquired during repetitive manipulation of objects or tools spanning decades, according to personal habitus (sport, arts, and work related) (Bril, 2015). Expertise is not only characterized by skillful execution of the tasks with optimal precision and timing, but also by fluidity, adaptability, versatility, and understanding of contextual affordances that are hardly summarized by tests outside the particular domain of expertise [review in Causby et al. (2014)]. According to Bernstein (1996) we shall consider that dexterity does not specifically refer to movement of the hand and fingers but more globally to the ability to engage the whole upper-limb in seamless interaction with tools or other objects appropriated from that person’s environment.

After stroke, the impairment of hand function largely depends on the location and extent of the brain lesion. While the physiological basis of movement deficits consequent to a lesion in the pyramidal tract is well demonstrated, specific mechanisms contributing to optimal recovery are less apparent. Indeed, the condition presented by any patient several months after stroke results from the direct consequences of the lesion alleviated by potential compensatory mechanisms developed by the patient in reaction to the impairment (Roby-Brami et al., 2003a; Levin et al., 2009). Various upper limb interventions (motor learning methods, neurostimulation techniques, robotics, virtual reality and serious games) are associated with improvements in motor performance, but many patients continue to experience significant limitations with object handling in everyday activities. Exactly how we go about consolidating adaptive motor behaviors through the rehabilitation process thus remains a considerable challenge. An important part of this problem is the ability to successfully distinguish the extent to which a given gesture is determined by the neuromotor impairment and by a compensatory mechanism. This question is particularly complicated in tasks involving manual dexterity where prehensile movement is contingent upon the task (individual digit movement, grasping, manipulation…) and its objective (placing, two step actions…), as well as personal factors (motivation, acquired skills, life habits…) and contextual cues related to the environment (presence of tools or assistive devices…).

However, in routine clinical examination, there is still a lack of comprehensive integrative methods capable of distinguishing the direct consequences of neuromotor impairment from compensatory strategies. The clinical evaluations of upper-limb function after stroke is based on several tests, organized according to the International Classification of Functioning (ICF). The global motor impairment is often measured using the Fugl-Meyer assessment (FMA) which examines movement speed, force, and range of motion through the upper limb, as well as the impact of abnormal synergies on voluntary actions (Fugl-Meyer et al., 1975). It is complemented by measures of spasticity (Ashworth). There exist numerous tests to quantify hand and upper-limb activity, most using the manipulation of objects such as Action Research Arm Test (ARAT), Jensen, Wolf Motor Function Test (WMFT) [see references in the reviews (Alt Murphy et al., 2015; Santisteban et al., 2016; Villepinte et al., 2020)]. In general, these tests give a unique score reflecting the level of success across a series of items, with penalties attributed where directives regarding timing or movement quality are not respected. Alterations of upper-limb dexterity is a significant cause of activity limitations that can be evaluated using the Motor Activity Log (MAL) which is a questionnaire investigating how frequently and how well the patient uses his/her affected upper limb at home (Taub et al., 1993). Most available methods for the evaluation of dexterity do not afford the description of compensatory strategies, with the exception of one recent proposition integrating observational kinematics to appreciate the quality of movement coordination (Alouche et al., 2020).

In this text, we shall question the link between consequences of brain lesions, functional impairments, motor strategies and performance in activity, particularly during object handling tasks. The presentation will focus on quantitative kinematic and kinetic studies, since we assume that routine use of quantitative methods should provide greater insight into compensatory motor behavior in stroke patients, in agreement with recent international consensus (Winstein and Varghese, 2018; Kwakkel et al., 2019). On a theoretical level, the perspective provided here draws upon the physiological basis of human motor control and concepts developed in ecological psychology. Following this logic, we consider that motor behavior emerges through interactions and reciprocal constraints defined by the specific task parameters, the individual’s sensorimotor attributes and the configuration of the environment (Newell, 1986). In a clinical context, this suggests that patients gravitate toward certain motor strategies adapted to their own specific condition (Latash and Anson, 1996) through action-perception cycles (enaction). This description of dexterity is consistent with the theoretical framework of Embodied, Embedded, Enactive, and Extended cognition (4E perspective) suggesting that the shape of individual motor strategies is embodied (dependent of bodily constraints), embedded into the environment (e.g., home), enacted (built through interaction) and potentially extended by assistive devices (Rowlands, 2010). According to these approaches, we shall consider that factors influencing human dexterity extend much beyond the ability to move fingers or to handle simple objects. Indeed, daily life activity is both the final outcome of post-stroke rehabilitation and a means in itself as part of a comprehensive rehabilitation program.

In this text, we examine those features of upper limb function and manual dexterity which are direct consequences of the brain lesion and those which result from compensation. Our objective is to explore how these contrasting processes emerge during the course of neurorehabilitation. We shall examine the consequences of brain lesions and the process of compensation at three integrative levels: biological (brain plasticity and vicariance), elementary sensorimotor function (sensorimotor patterns) and activity (acquisition of new motor strategies for object reaching and handling) the two first levels together refer to ICF “body functions and structure.” In particular, we insist on the contribution of quantitative kinematic and kinetic methods for improved understanding of these recovery and compensation dynamics. Thereafter, we shall discuss some clinical implications for rehabilitation and some perspectives, with a specific focus on the use of novel technology in this field.

## Biological Change to Upper Limb Motor Pathways Following Stroke

### Neural Control of Dexterous Upper-Limb Function

Through the course of the 20th century, clinical studies documented how lesions to a given cerebral hemisphere induced contralateral hemiparesis, suggesting that the descending (pyramidal) tract projected to the opposite side of the body (Dejerine, 1914). Subsequent neuroanatomical studies showed that control of contralateral hand movements in macaque monkeys was directly dependent on the integrity of the descending corticospinal tract (CST) between the primary motor cortex M1 and the spinal level (Lawrence and Kuypers, 1968). Later, direct monosynaptic connections between M1 pyramidal neurons onto the spinal motor neurons were identified in monkeys (Muir and Lemon, 1983), then demonstrated in humans (Lemon et al., 2004). This descending monosynaptic pathway to the distal extremity is specific to primates (particularly humans and apes) capable of precision grip involving fingers and an opposable thumb. In lower mammals, descending motor commands for reaching and grasping are mediated via propriospinal and segmental interneurons [review in Alstermark and Isa (2012)]. While this latter system has also been demonstrated in humans (Pierrot-Deseilligny, 1996), its specific role in human prehension remains to be determined (Giboin et al., 2012). Beyond the monosynaptic CST pathway, there are multiple motor brain areas, in each hemisphere which, have descending projections contributing to motor tasks involving the whole upper-limb [review in Kollias et al. (2001)].

Hand dexterity and individual finger movements are critically dependent on somatosensory feedback provided by the primary somatosensory area or thalamus, as demonstrated in monkeys (Asanuma and Arissian, 1984). More generally, the control of motor behavior is distributed in a wide parieto-frontal network. The cognitive control of action is hierarchically organized in the frontal lobe (Koechlin and Jubault, 2006), with skilled action sequences orchestrated via the premotor cortex (Ohbayashi et al., 2016). Multisensory feedback, including visual and proprioceptive cues are integrated in the parietal lobe during goal directed behavior (Battaglia-Mayer et al., 2003), and ultimately projected back to the premotor cortex to inform the consequent gesture.

In brain injured patients after stroke, lesions generally extend beyond the CST, involving sensory and/or integrative brain areas. Somatosensory impairments linked to thalamic or parietal cortical lesions also significantly impair manual dexterity (Hermsdorfer et al., 2004; Meyer et al., 2016). The occurrence of neuropsychological syndromes such as apraxia or neglect remain outside the scope of the present review.

### Role of the Corticospinal Tract in the Impairment and Spontaneous Recovery Post Stroke

In neurological clinics, the pathological condition directly related to M1 or CST lesions is referred to as upper motor neuron syndrome. Typically, this involves negative symptoms through the distal upper extremity with weakness of the hand and fingers resulting in decreased force, speed and range of motion as well as the loss of individuated finger control. At the same time, patterns of abnormal muscle overactivity such as spasticity due to increased excitability of the stretch reflex provoke spasticity (Burke et al., 2013; Levin, 2016) and muscle co-contraction (Gracies, 2005b) that can result in soft tissue contractures (Gracies, 2005a). Many recent studies documented the link between the lesion of the CST and the severity of the hemiparesis using brain imaging with tractography or electrophysiological techniques such as transcranial magnetic stimulation (TMS) (Lindberg et al., 2007; Buetefisch et al., 2018; Birchenall et al., 2019). The ability to make selective finger movements proves to be dependent on CST integrity (Lang and Schieber, 2004; Birchenall et al., 2019).

After stroke, hemiparesis may spontaneously recover during the subacute period, however, this recovery is inconsistent and usually incomplete, plateauing generally after a few months (chronic stage). An influential theory proposes that spontaneous recovery represents a fixed proportion, around 70%, of each patient’s maximum possible improvement (Prabhakaran et al., 2008). This rule has been confirmed in a variety of clinical conditions but its meaning remains disputed (Hope et al., 2019; Senesh and Reinkensmeyer, 2019). In effect, several studies show that certain patients do not necessarily obey this proportional rule for spontaneous recovery. Byblow et al. (2015) demonstrated that these outliers were characterized by severe alterations of the CST, demonstrated by anomalies of the motor potentials evoked by TMS. This suggests that the spontaneous recovery is heavily dependent on the restauration of neural tissue contributing to the ipsilesional corticomotor pathways.

Activity dependent brain plasticity has been demonstrated in animals for the primary motor area (Nudo et al., 1996). In addition, animal studies have demonstrated that after stroke, the plastic reorganization of the descending corticomotor pathways also involved the premotor and supplementary motor areas (Dancause, 2006). This has been confirmed by brain imaging studies in humans showing that recovery was associated with activation in ipsilesional medial-premotor and primary motor cortices [meta-analysis in Favre et al. (2014)].

**FIGURE 1:**
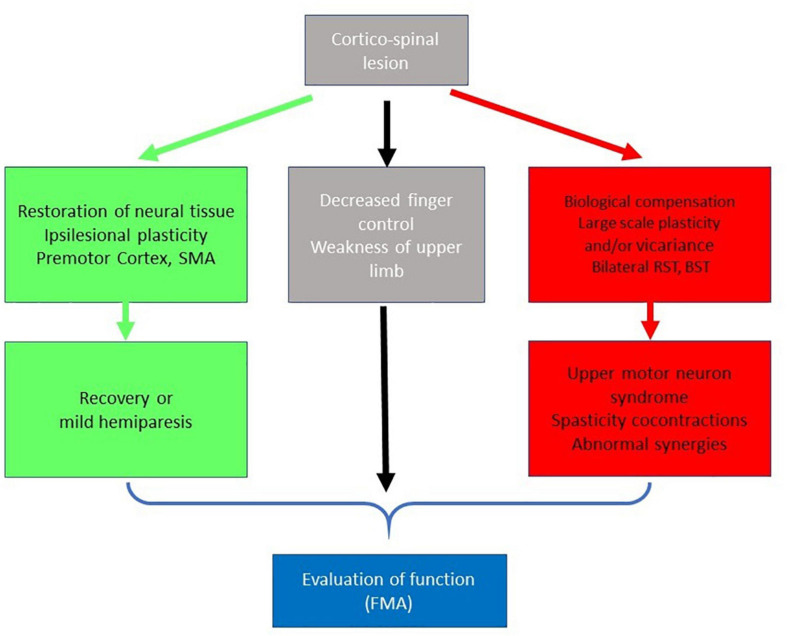
Simplified schema on the role of the Corticospinal tract in recovery. The extent of the CST lesion has direct consequences on dexterous upper limb control following stroke (gray squares). Spontaneous biological recovery can occur (green squares) via restoration of neural tissue or ipsilesional plasticity involving the premotor cortex and/or supplementary motor areas. Following a severe lesion, compensation for diminished CST integrity (red squares) may occur via large scale plasticity and vicariance involving polysynaptic bilateral pathways, including the reticulospinal and bulbospinal tracts. In this event, functional recovery is associated with maladaptive motor symptoms (upper-motor neuron syndrome). The level of impairment resulting from the combined effects of the CST lesion and neuroplastic change can be measured using motor performance tests such as the Fugl Meyer assessment (blue square).

Thusly, recovery at the biological level, in particular with respect to the ipsilesional CST, is an important vector for the return of upper limb motor control. At the same time, the neurological insult may give rise to a host of other vicariant biological structural processes involving the premotor and supplementary motor areas (Dancause, 2006) which may actively work to compensate for the deleted fibers of the pyramidal tract in order to contribute to the functional recovery of dexterity. Figure 1 provides a schematization of this recovery process, indicated in green.

### Compensation Through Large Scale Vicariance and Neural Plasticity

After severe lesions of the CST, larger scale plasticity and excitability changes can result in alternative pathways being solicited across sensorimotor networks in both hemispheres (Xerri et al., 2014; Baker et al., 2015; Bundy and Nudo, 2019). This bilateral reorganization may sustain a certain level of upper-limb function, notably via reticulospinal and rubrospinal tracts (Belhaj-Saif and Cheney, 2000; Zaaimi et al., 2012). In clinical observations, a unilateral lesion of the CST induces bilateral impairment of upper-limb function. This is likely due to the presence, and consequent impairment, of non-decussating pathways to the ipsilateral segments (Desrosiers et al., 1996). Similar large scale bilateral reorganizations are also demonstrated in clinical studies (Gerloff et al., 2006; Baker et al., 2015; Buma et al., 2016; Peters et al., 2017). The involvement of alternative ipsilesional and contralesional brain areas relaying at subcortical level in the reticulospinal and rubrospinal tracts has been demonstrated in humans (Honeycutt et al., 2013; McMorland et al., 2015; McPherson et al., 2018). The specific role of the contralesional hemisphere is particularly disputed. On one hand, it could contribute to the recovery of the affected side thanks to ipsilateral cortico-spinal pathways (Dodd et al., 2017). On the other hand, it can have negative effects on the lesioned hemisphere via increased transcallosal inhibition (Murase et al., 2004). The reticulospinal system, which is activated bilaterally, contributes to motor function on the hemiparetic side, as shown by starling reactions, but this phenomenon is negatively correlated with hand dexterity (Choudhury et al., 2019). The alternative descending motor pathways converge on propriospinal relays which appear more heavily engaged in transmission of motor commands after stroke (Mazevet et al., 2003). The rearrangement of neuronal pathways due to plasticity and excitability changes has maladaptive effects. In particular, pathological muscle synergies, spasticity and excessive co-contractions are likely due to the exaggerated involvement of the reticulospinal (Li, 2017; McPherson et al., 2018) and/or bulbospinal tracts (Owen et al., 2017). For example, increases in fractional anisotropy in these pathways appears to be correlated with the severity of pathological upper limb synergies and hand impairment (Owen et al., 2017).

In brief, when the monosynaptic CST is severely lesioned, fine motor control and hand dexterity are compromised but large-scale changes in brain networks with multiple relays may assure a certain level of motor function at the cost of maladaptive phenomena. This process is indicated in red in Figure 1.

## Impairments in Dexterous Coordination of the Upper Limb Post Stroke

Broadly speaking, patients with mild to moderate hemiparesis retain the global spatial organization of prehensile movement in the context of goal directed reaching, multi-finger actions during grasp and grip-load force coordination. Nonetheless, the neurological effects of the cerebrovascular accident bring about various irregularities in upper limb kinematics and dexterous control of the hand.

A pathophysiological analysis is necessary in order to better understand the mechanisms of improvement in function, and decipher compensation from true recovery. This means that clinical symptoms should be interpreted in the light of the physiology of the motor system. In the following section, we will focus on quantitative movement analysis (kinematics and kinetics) during well-defined elementary upper-limb tasks (Lemon, 1999; Nowak and Hermsdorfer, 2006; Kwakkel et al., 2019). These experimental paradigms which represent prototypes of naturalistic hand movements have been studied extensively in both healthy subjects and stroke patients.

### Kinematics of Reach to Grasp Movements

#### Physiological Control of Goal Directed Movements

Pointing tasks involve the displacement of the hand, or working point of the upper limb toward an object of interest. In healthy subjects, this movement tends to be predominantly mediated via feed-forward control, as evidenced by the smooth, bell-shaped velocity profile described by the hand (Abend et al., 1982). Prehension tasks couple reach and grasp components, identified by the pioneering works of Jeannerod (1984). The pre-shaping of the finger aperture during reaching and the smooth peak velocity of the reaching hand show that control is anticipated as a function of the object position in space and of its intrinsic characteristics [review in Jeannerod (2009) see Smeets et al. (2019) for an alternative interpretation of the coupling]. In these types of prehensile tasks, the upper-limb may be seen to possess no less than 7 degrees of freedom (DoF) afforded by the rotational axes of the shoulder, elbow and wrist. The pointing task is defined by 6 DoF, corresponding to the 3D position and orientation of the object in space. As a result, there is kinematic redundancy at the level of the upper limb as the 7 DoF all contribute to the displacement of the hand for grasping in the 3D space (Desmurget and Prablanc, 1997). As initially proposed by Bernstein (Bernstein, 1967), control of the upper limb may be based on synergies which share the same spatio-temporal properties, and can be additively combined in a task specific way. There is still no agreement as to whether synergies are coordinated at the joint kinematic (Scholz et al., 2000; Yang et al., 2002) or muscular level (d’Avella and Bizzi, 2005; Ting and McKay, 2007; Bizzi et al., 2008). The Uncontrolled Manifold theory subscribes to the former, suggesting that synergies are flexible and allow automatic compensation between elements in order to stabilize the important task related variables such as the displacement of the endpoint (Yang et al., 2007; Latash, 2008; Martin et al., 2009). As proposed by Feldman, the neural basis of motor control, bridging the gap between physiology and biomechanics, could be the modulation of the stretch reflex threshold by the CST acting on motor neuron membrane potential (Feldman, 2015), consistent with the formation of synergies (Latash et al., 2010). This theory is disputed, and the reduction of redundancy by synergies has also been interpreted in the framework of optimal control (Guigon et al., 2007). However, there is accepted evidence that the primary motor cortex is responsible for the coordination of muscle and joints to generate the spatio-temporal form of goal directed movements (Kalaska, 2009; Capaday et al., 2013).

#### Movement Features Consecutive to CST Lesion or to Compensation in Stroke Patients

**FIGURE 2:**
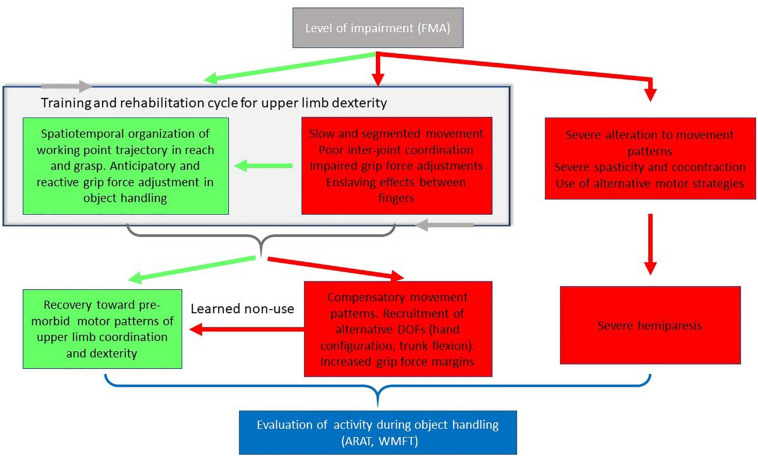
Simplified schema of the consequences of the impairments on movement features. In mild to moderate motor impairments, patients may or may not retain the ability to regulate working point trajectory in reach and grasp, or to effectively adjust grip force during object handling. These spatiotemporal and kinetic parameters evolve through the course of training and rehabilitation (cycle indicated by gray arrows). Inter-joint movement coordination may either progress toward premorbid patterns of motor control (green arrows) or toward compensatory movement patterns involving the recruitment of alternative degrees of freedom to compensate for the impairment (red arrows). Compensatory changes may inhibit progress toward pre-morbid coordination due to the learned non-use phenomenon. In the case of severe impairment, control of spatiotemporal organization of upper limb movement is perturbed, with functional tasks and actions (when they are possible) executed with alternative strategies. Evaluation of activity during object handling may be carried out using clinical tools including the Action Research Arm Test (ARAT) or Wolf Motor Function Test (WMFT).

After stroke, patients generally exhibit decreased force and range of motion across joints with alterations in movement coordination, referred to as pathological synergies (Brunnstrom, 1970). Kinematic analysis underscores these direct consequences of the CST lesion and may equally serve to distinguish certain compensatory mechanisms (Figure 2).

The most prominent aspect of the CST lesion on kinematics is the duration of upper limb movements, with observable segmentation of the velocity profile (Trombly, 1993; Roby-Brami et al., 1997; van Dokkum et al., 2014; van Kordelaar et al., 2014; Buma et al., 2016). Smoothness metrics, such as the jerk, or spectral arc length, may be used to quantify this particular kinematic feature. This may provide an indication of the level of motor control, or ability to perform efficient movements (Rohrer et al., 2002; Balasubramanian et al., 2015; Buma et al., 2016). Through the course of stroke recovery, reduced smoothness might suggest problems in the central blending of sub-movements (Rohrer et al., 2002). Another hypothesis is that smoothness deficits reflect the suboptimal performance of secondary sensory-motor brain areas (Buma et al., 2016) recruited at the structural level to compensate for the faster and more synchronized CST.

Secondly, the normal flexible shoulder-elbow coordination is disrupted with anomalies in synergistic control at both joint (Levin, 1996; Reisman and Scholz, 2003; Roby-Brami et al., 2003b; Micera et al., 2005) and muscular levels (Beer et al., 2000; Cheung et al., 2012). The normal flexible inter-joint coordination is replaced by stereotypic synergies (Twitchell, 1951; Dewald and Beer, 2001). As a result, hand orientation at the time of grasping is also altered (Roby-Brami et al., 2003b; Sangole and Levin, 2009). It is likely that the disruption of the normally flexible kinematic and muscular synergies, is a direct consequence of the lesion of the primary motor area (Capaday et al., 2013). Abnormal stereotyped synergies are probably due to the reorganization of the neural pathways at the structural level, as described in the Section “Biological Change to Upper Limb Motor Pathways Following Stroke.” Changes in shoulder flexion and elbow extension post stroke limit the upper-limb workspace, and consequently the patient’s reaching abilities (Levin et al., 2002; Reisman and Scholz, 2003; Roby-Brami et al., 2003a). However, hemiparetic patients with mild impairment may retain the ability to use the abundance of DoF to stabilize the trajectory of the hand via automatic compensation of errors between DoF (Reisman and Scholz, 2003). This ability is less flexible than in healthy subjects, particularly when the trunk is involved to reach more distant targets (Reisman and Scholz, 2006).

In effect, coordination between the upper limb and trunk is modified, and stroke patients make excessive use of trunk flexion in forward reaching tasks (Roby-Brami et al., 1997; Cirstea and Levin, 2000; Levin et al., 2002). This is interpreted as a compensatory movement pattern in response to the shortening of the functional length of the limb, arising from impaired elbow extension (Cirstea and Levin, 2000; Roby-Brami et al., 2003a). Indeed, this voluntary control of the trunk remains relatively preserved in most stroke patients due to its bilateral control (Robertson and Roby-Brami, 2011). According to the context, patients may keenly adjust trunk rotation to target direction (Robertson and Roby-Brami, 2011) and to the possible voluntary triggering of pathological synergies (van Kordelaar et al., 2012; Levin et al., 2016). The involvement of the trunk in reaching is interpreted as the spontaneous adaptive use of the redundant and abundant DoF of the body allowing task accomplishment (displace the hand) despite the upper-limb impairment. The use of such compensations may, however, mask a patient’s actual movement abilities. Providing specific instructions to a patient, or use of a mechanical constraint to limit compensatory movement patterns can result in qualitatively better performance. For instance, when the trunk is blocked, a reaching movement within the arm workspace can be successfully executed (at greater effort) with an improved shoulder-elbow coordination (Michaelsen et al., 2001; Levin et al., 2004; Bakhti et al., 2018). Preference for compensatory movement patterns though, do risk becoming highly automated in what is called “learned non-use” (Taub et al., 2006), (Figure 2, also see section “Manipulation and Object Handling”). Once established, these behavioral changes may be difficult to break down, and limit long term clinical outcomes.

### Independent Finger Control

The possibility to perform individuated finger control is important for human hand dexterity, with a maximum precision and mobility for the thumb (Marzke and Marzke, 2000). The selectivity of finger control in healthy subjects is not perfect since the action of one finger involuntarily activates others during force production (Zatsiorsky et al., 2000) or kinematic tasks (Teremetz et al., 2015).

Loss of strength and individuated finger control is common following stroke. Generally speaking, both appear to be associated with the integrity of the CST (Wolbrecht et al., 2018; Birchenall et al., 2019). Even after substantial recovery of a pure motor hemiparesis the individuation of finger movements remains limited, particularly in finger abduction (Lang and Schieber, 2003, 2004) due to the lack of selective muscle activation (Schieber et al., 2009; Kamper et al., 2014). Electromyography coupled with grip force measures demonstrate irregular patterns of muscle activation which limit the ability to generate forces and regulate directional control of the fingers and thumb (Cruz et al., 2005; Seo et al., 2010; Triandafilou et al., 2011). Further to this, exaggerated enslaving effects between fingers and the thumb have been observed in studies using both kinematic (Raghavan et al., 2006) and kinetic (Li et al., 2003) measures. This increased involvement of the extra digits is consistent with the fact that stroke patients tend to have relatively important activation of long finger flexors during the generation of fingertip flexion forces compared to healthy control subjects (Cruz et al., 2005).

Nonetheless, individuated finger control appears only weakly correlated with clinical measures of hand dexterity based on grasping actions, suggesting different cortico-spinal control modes of the fingers according to the task (Raghavan et al., 2006).

### Hand and Finger Configuration for Grasping

The redundancy of the human hand and fingers with 22 DoF allows to adapt the global hand configuration to the shape of various objects. Principal component analysis of finger joint rotations during grasping and reaching to grasp demonstrated that the DoF were coupled in additively combined synergies: the first component corresponding globally to opening-closing of the hand, with the others contributing to finer adaptation to the shape of the object (Santello et al., 1998). These synergies are progressively formed during reaching, confirming that hand configuration is preshaped as a function of object characteristics (Santello and Soechting, 1998) and task related constraints [reviews in Bicchi et al. (2011) and Santello et al. (2016)]. The curvature of the palmar arch is also preshaped during reaching (Sangole and Levin, 2009). As for proximal upper-limb synergies, hand synergies are probably generated at the cortical level, particularly within the primary motor and somatosensory areas (Leo et al., 2016).

After stroke, the kinematics of the grasping component show impairments and delays in the palmar arch modulation and finger pre-shaping in preparation for grasping (Sangole and Levin, 2009; Tretriluxana et al., 2009). The kinematics of the grasp aperture opening is slow, jerky and less precise with increased delay (Lang et al., 2005; van Kordelaar et al., 2014).

Hand and finger gestures also benefit from compensatory mechanisms. Since performance in precision grip is not completely correlated with the independent control of the fingers, Raghavan et al. (2006) suggested the intervention of compensatory mechanisms within the cerebral sensorimotor networks for grasping actions. This was subsequently demonstrated by a kinematic study of finger joint rotations during reaching to grasp objects with different shapes (concave or convex) (Raghavan et al., 2010). They observed that patients were able to perform different finger coordination to adapt to object shape despite the reduction in finger abduction, PIP (proximal interphalangeal joint) flexion and MCP (metacarpophalangeal joint) extension. The compensatory coordination involved MCP flexion in a later stage of reaching to compensate for the reduction in MCP flexion and to adapt grasp aperture to the shape of the object (Raghavan et al., 2010).

### Force Exchanges During Interactions With Objects

A complementary approach for the analysis of manual dexterity is the quantification of force exchanges between the hand and a given object. The physiology of precision grip was pioneered by Johansson and co-workers who used a handheld device equipped with force sensors and an accelerometer to analyze the control of a lifting task (Westling and Johansson, 1984). These, and subsequent studies by other teams demonstrate how grip and load forces increase in parallel prior to lifting the object. In healthy adult subjects, the magnitude of these grip forces is precisely adapted to the anticipated characteristics of both the object (weight, size, shape, and frictional characteristics) and the dynamics of the task. During displacement of the handheld object, grip force is maintained above a safety margin, preventing accidental slippage [reviews in Johansson and Cole (1992) and Flanagan et al. (2009)], and continually adjusted proportional to the load forces associated with the mass and acceleration of that object (Wing, 1996; Hermsdorfer et al., 2003). Each contact event during the performance of the sub-goals of the grasp to lift task is signaled by a distinct sensory event and any perturbing event can be rapidly corrected thanks to short latency cortical loops involving the somatosensory and primary motor areas [reviews in Johansson and Flanagan (2009)].

Hemiparetic patients with moderate disability (Hermsdorfer et al., 2003; Nowak et al., 2003; Quaney et al., 2005; Nowak and Hermsdorfer, 2006) and children with CP (Eliasson et al., 1992; Duque et al., 2003; Bleyenheuft and Thonnard, 2010) tend to exhibit grip force adjustments following the general movement dynamics [review in Bleyenheuft and Gordon (2014)]. This suggests that patients with unilateral brain lesions retain predictive anticipatory motor control in precision grip tasks, which facilitates cyclic movements in particular. During discrete movement (e.g., grasp and place) however, time delays between maximal grip force and load force are often in excess of 200 ms. Temporal irregularities and excessive time delay could be due directly to the CST lesion (Duque et al., 2003) or to delays within transcortical responses. These latter observations suggest that lesions to cerebral sensorimotor networks limit the ability of the patient to reactively modulate grip forces with respect to complex or unexpected movement dynamics (Hermsdorfer et al., 2003).

At the same time, hemiparetic patients typically present with an increased magnitude of grip forces across these different tasks [reviews in Nowak and Hermsdorfer (2009) and Bleyenheuft and Gordon (2014)]. This disruption in grip to load force ratio is similarly observed in healthy individuals immediately following digital anesthesia (Nowak et al., 2001; Monzee et al., 2003). This increased grip force is thus considered to be a highly automated compensation whereby the safety margin is increased to prevent slippage in the context of impaired sensation.

In brief, certain features expressed in most functional movements are likely the direct consequence of the lesion to the CST and/or somatosensory cortex (i) loss of individual finger movement, (ii) general slowness with temporal irregularities and jerkiness, (iii) alterations of the somato-motor reactive control with time delays, (iv) disrupted inter-joint synergies in the upper-limb and the hand. The analysis of hand and finger function in stroke patients illustrate the complex role of the primary motor cortex, both excitatory for the generation of synergies, and inhibitory for the selective activation of muscles. Other pathological features such as abnormal movement synergies are likely due to maladaptive plasticity as described in the Section “Compensation Through Large Scale Vicariance and Neural Plasticity.” Compensation at the level of motor patterns is evidenced by the spontaneous use of extra DoF in the trunk and the finger joints and by the increased level of force to increase the safety margin.

## Naturalistic Activity: Compensatory Strategies to Ensure Task Completion

**FIGURE 3:**
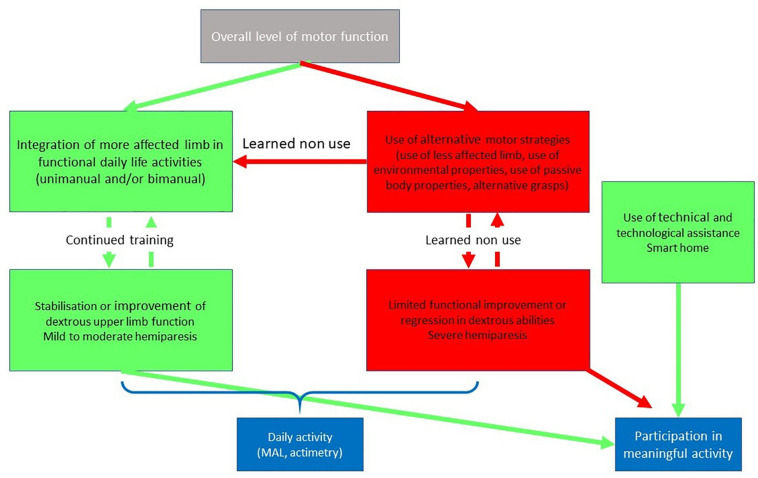
Simplified schema of the relations between the overall level of motor function and daily activity. If the motor function allows the use of the most affected limb in daily life (mild or moderate hemiparesis), the improvement acquired by training is stabilized. If a patient with moderate to severe hemiparesis develops alternatives motor strategies, such as the use of the less affected limb, there is a risk is of functional decline due to the learned-non use phenomenon (i.e., “Use it and improve it or lose it”). Integration of the affected upper limb in functional daily life activities may be measured using questionnaires, such as the Motor Activity Log (MAL), or sensor based actimetry parameters. In case of severe hemiparesis, technical and technological assistance may compensate for upper limb deficits and support participation in meaningful activity.

Dexterous upper-limb function is particularly important for the performance of daily activities, which frequently involves the manipulation of objects and tools. The gestures used for naturalistic actions in ecological contexts are more sophisticated than simple prototypic gestures, with a great variability and considerable interindividual differences. When the impairment is too severe and functional sensorimotor adaptations (as described in section “Impairments in Dexterous Coordination of the Upper Limb Post Stroke”) are overwhelmed, a patient may be unable to perform a given action in a habitual or spontaneous manner. In order to carry out said action, they may, however, voluntarily adapt a given motor plan or develop alternative strategies in order to satisfy task objectives. In a simplified and operational way, we shall consider that a motor plan is defined by the trajectory of the working point of the limb. Alternative strategies can thus be executed with alternative working points and/or different trajectories. Consistent with Newell’s ecological framework (Newell, 1986), this can be achieved by adapting the characteristics of the task and/or the disposition of resources in that environment (Figure 3).

### Task Related Alternative Strategies

The impact of alternative strategies on dexterous upper-limb function will be presented for three representative tasks: unimanual object grasping, manipulation and bimanual activities.

#### Contrasting Patterns in Reach and Grasp Actions

Analytical studies of hand dexterity focused on precision grip or reach to grasp actions have generally overlooked the great variety of hand configurations adapted to the multiple tasks of everyday life (Bullock et al., 2013). The great flexibility of the human hand to grasp objects (Kapandji, 1980) has been mainly studied using qualitative methods. Napier (Napier, 1956) proposed a dichotomous classification of grasping: “precision grip” and “power grasp.” Later, Iberall et al. (1986) added an intermediate “key grip” and proposed a systematic description of possible finger opposition configurations. This taxonomy of grasping was further developed in the context of anthropomorphic robotics (Feix et al., 2015).

The impairments due to stroke limit the possibilities of action on the environment and, as a consequence, the hemiparetic patient may develop alternative prehension strategies. In order to experimentally investigate this compensation phenomenon, patients must be free to act spontaneously, independent of unnecessary physical constraints or external instruction. Kinematic studies of reach to grasp indicate considerable differences in motor planning. In a study by Roby-Brami et al. (1997), two notable reaching patterns were distinguished. The first involved relatively direct movement of the hand along the sagittal axis toward the object, using the table as a support for the weight of the hand during reaching, this was referred to as a “sliding” strategy. The second pattern observed in this study was characterized by a greater amplitude of vertical movement as patients lifted the hand and descended upon the target object, an action described as “grasping from above” [similar patterns are also observed in tetraplegic patients; (Laffont et al., 2000)]. Importantly, each of these prehensile actions were found to be associated with the severity of hemiparesis, with the “sliding strategy” observed in patients with more proximal weakness and “grasping from above” in those with more distal weakness. These observations illustrate how distinct changes in motor planning emerge in response to the specificity of the motor impairment. Additionally, the ‘sliding strategy’ provides a simple example of how patients may spontaneously exploit features in the environment in order to carry out functional tasks in naturalistic activity.

Significant variation in grasp configuration is also observed in patients post-stroke. When displacing handheld objects, healthy subjects generally use a precision grip or multipulpar grasp including the thumb and a number of fingers according to the size of the object (Cesari and Newell, 1999). Stroke patients, however, appear to use these particular grasp configurations much less frequently when employing objects regularly used in daily life activities (Roby-Brami et al., 1997; Bensmail et al., 2010; Garcia Alvarez et al., 2017). For example, while the majority of healthy subjects used multipulpar grasps to take a spoon, water bottle or ball, the different stroke patients used various combinations of palmar and digito-palmar grasp configurations (Garcia Alvarez et al., 2017). Others still were seen to use a particular “raking” strategy, either with the four fingers and the palm parallel to the table, or with the ulnar aspect of the hand, the palm perpendicular to the table.

Again, the movement variability observed in these works are likely associated with the individual’s impairment and the means by which that person elects to overcome the associated movement limitation. More specifically, we propose that the preservation of precision grasps in some patients might be attributed to less severe lesions of the pyramidal tract or recovery due to cortical plasticity. In contrast, alternative “raking” grasp strategies could be archaic motor acts similar to those of monkeys who lack thumb opposition and have less developed cortico-spinal tracts (Maier et al., 2005). This interpretation is consistent with Jackson’s dissolution concept (York and Steinberg, 1995) and the hierarchical evolutionary organization of dexterity proposed by Bernstein (Bernstein, 1996). Second, we suggest that patients preferentially use standard grasp types if they can. Increased severity of the motor deficit will consequently limit the range of grasp-types possible for that patient, with the ultimate selection of a given hand configuration reflecting the specificity of their impairment (e.g., fine thumb control, spasticity, limitation of the thumb-forefinger opening …).

Finally, we propose that there are causal interactions between impairment and compensation at proximal (shoulder and elbow) and distal (hand and fingers) levels that determine the pose of the hand at the time of grasping. In hemiparetic patients, the hand is oriented more frontally, inclined downward with a variable axial rotation than that which is typical in healthy subjects (Roby-Brami et al., 2003b). Indeed, if a patient uses trunk compensation (flexion and internal axial rotation) or pathological patterns of shoulder and elbow coordination, the hand will be mechanically inclined downward, further constraining potential grasp configurations. Conversely, alternative grasping strategies may impose specific motor planning with atypical hand position and orientation relative to the object and, by consequence, an atypical reaching trajectory inducing deviant kinematic features. These close and complex functional interactions between the proximal and distal parts of the upper-limb may explain that, while cortico-spinal control is mainly distal (Maier et al., 2005), grasping is not more functionally impaired than reaching (Lang et al., 2005). The evolution of these prehensile compensatory strategies may account for the poor correlation between independent finger control and clinical tests of hand function in stroke patients (Raghavan et al., 2006).

#### Manipulation and Object Handling

In contrast to prehensile tasks aiming at stabilizing the object relative to the hand, manipulation requires the ability to move and rotate the object relative to the body, and supports the use of tools for actions on the environment. Manipulation imposes greater challenges to the sensorimotor system than grasping, including the anticipation of inertial forces and torques in response to variations in the position and orientation of the handheld object (Schneider et al., 2020). In-hand manipulation implies a particularly sophisticated form of manual dexterity where independent finger movements enable the displacement of the object with respect to the hand (Elliott and Connolly, 1984). A classification system has been adopted in order to describe a variety of actions during naturalistic activity in professional or household contexts (Bullock et al., 2013) as well as the use of prosthetic devices by amputees in their homes (Spiers et al., 2017). In daily life activities, performance of object handling tasks is conditioned by the characteristics of the individual and the disposition of the environment. Given the unstructured nature of daily life activity, effective motor solutions may be generated using any number of action sequences and postural configurations. Moreover, certain regularities in grasp transitions (e.g., top grasp to power grip) have been observed in daily life activities (Bullock et al., 2013), suggesting that use of a given hand configuration influences the subsequent prehensile activity. Even for highly repetitive assembly tasks, considerable variability in hand gestures can be observed across different actors (Brunet and Riff, 2009).

Generally speaking, the quantitative analysis of manipulation tasks in stroke patients has received relatively limited attention. Kinematic studies of drinking movements are one exception to this, and recommendations exist on the standardization of this task in order to provide reproducible clinical data (Alt Murphy et al., 2012; Kwakkel et al., 2019). Movement variables examined in these contexts remain, nonetheless, similar to those examined in reaching tasks, including movement duration, hand velocity and smoothness (Alt Murphy et al., 2012). Predictably, stroke patients with poor upper limb function tend to present with segmented velocity profiles and greater total duration for performance of the drinking task (Alt Murphy et al., 2012).

More recently, studies incorporating instrumented objects for measuring force exchanges during tasks involving object rotation have begun to provide additional perspective (Hermsdorfer et al., 2003). Our team designed an instrumented object to be easily manipulated by a person with an upper-limb movement disorder (Jarrassé et al., 2013). This device facilitates the analysis of the sequence of phases involved in object manipulation tasks (Martin-Brevet et al., 2017). Moreover, this method may also serve to highlight micro-errors occurring in action performance at the transitions between sub-goals (Seligman et al., 2014). For example, when using their hemiparetic arm, stroke patients experience greater difficulty with maintaining the vertical orientation of the handheld object, most notably in the transitions to/from a table (i.e. object lifting and object placement) (Parry et al., 2019). In addition, measurable “touch” and “push” errors observed in the form of force variations on lateral load cells prior to establishing grasp as well as increased downward force upon the object following placement. Ongoing research aims at characterizing how stroke patients perform object handling and regulate grip forces during prototypical rotational tasks (e.g., lifting a cup to the mouth, pointing a remote control).

#### Bimanual Activities

Of course, in everyday life, most activities require some form of bimanual coordination. Moving with cooperative spatiotemporal precision, both hands have differentiated and specialized roles (i.e. the left hand holds and orients an object while the right hand performs an action on it) (Kantak et al., 2017). However, while there is a reasonable body of literature on bimanual organization through infant development (Fagard and Lockman, 2005) and primate evolution (Obhi, 2004), there is a paucity of literature on bimanual cooperative actions during daily life tasks in human adults. Existing studies on the subject have tended to focus most notably on the role of executive functions in movement planning (Gulde et al., 2019).

Despite their clinical interest, bimanual gestures remain largely unexplored in hemiparetic patients (Haaland et al., 2012). It is well known that stroke impairs both sides of the body since the less affected, ipsilesional side, also presents with weakness (Colebatch and Gandevia, 1989) and reduced hand dexterity (Cunha et al., 2017), generally proportional to the severity of the hemiparetic impairment (Maenza et al., 2020). Differential effects upon the ipsilesional hand are observed according to the cerebral hemisphere involved. Most notably, right sided lesions incur problems with visuospatial aspects of coordinated prehensile gestures while left sided lesions have greater effects upon planning and sequencing of the action sequence (Hermsdorfer et al., 1999).

The functional role of the less affected limb of stroke patients is controversial. On one hand, the patients may tend to use their less-affected hand for daily life activity as a compensatory strategy. As stated previously, this preferential use of the less-affected limb may inhibit implication of the hemiparetic counterpart in functional activity and thereby hinder recovery (Taub et al., 2006; Hidaka et al., 2012). It is often assumed that the improvement of the contralesional paretic arm by active rehabilitation will help patients in their (bimanual) daily activities (Johnson et al., 2011). However, some results suggest a limited transfer from unimanual training to bimanual activity (Johnson et al., 2011). In functional assessment of the upper limb during instrumental activities of daily living (IADLs), the use of both arms together favors performance when compared to modal use of the hemiparetic or less affected upper limb (Haaland et al., 2012). As proposed by Haaland et al. (2012) “rehabilitation therapy should focus on the ipsilesional as well as the contralesional arm.”

Taken together, these principles underscore how dexterity after stroke should be broadly understood as a skillful way to perform purposeful actions, either unimanually by using alternative reaching and grasping strategies or bimanually. Beyond the ability to carry out prototypical gestures with the hand and arm, dexterous function is something which engages both upper-limbs and likely the whole body.

### Adaptation of the Environment to Support Activity

The ultimate objective of rehabilitation is to favor independence and quality of life for the patients in their living environment, be that in their own home or in a supported care facility. In this context, dexterity does not only represent the gestures of the hand but the individual’s capacity to act on his/her environment. The configuration of the environment may provide affordances that facilitate the behavior, either as hand-held assistive devices or using home automation solutions.

The evaluation of at-home occupational or multidisciplinary interventions is relatively recent [review in Wolf et al. (2015)]. Occupational therapy involves the provision of specialized tools or assistive devices that can support impaired dexterity and improve the functional independence of patients. There exists a variety of low-tech assistive devices (with adapted handles, cuffs, loops, reachers…) for all areas of self-care including dressing, bathing, grooming, cooking, feeding, toileting etc. In addition, readily accessible technology in contemporary home environments may serve as a mediator of actions. Interestingly, recent developments propose technological solutions integrating embedded sensors in various devices, and at various locations in domestic environments to guide and assist daily living activities of patients with diverse neuropsychological impairments (Worthington, 2016; Baber et al., 2017). Smart home systems have a large potential to compensate for limitations in dexterous upper limb function, promote participation and improve quality of life. However, a recent review pinpoints the lack of high-quality evidence supporting the use of such devices. Further to this, ethical concerns (e.g., privacy) and the importance of human contact in personal support packages remain important considerations (Jamwal et al., 2020). Specific methods are needed to better understand how patients with limited dexterity cope with common household tasks. Recent progress in sensor technology, particularly accelerometry, combined with novel signal processing methods are quite promising but remain less developed for applications in upper-limb movement analysis than for the lower limb or gait [review in Dobkin (2017)]. Accelerometry methods have been used to quantify the contribution of both upper-limbs to activity for each time unit during several hours (Bailey et al., 2015). They confirm the expected asymmetry due to hemiparesis and can document non-use of the affected side during bimanual activities (Michielsen et al., 2012). Thus, accelerometry methods afford particularly interesting possibilities for quantifying the effects of rehabilitation techniques on both limbs in ecological contexts (Wang et al., 2017), providing meaningful data on patient activity, which might be complementary to established clinical evaluation techniques (Bailey et al., 2015). However, further progress on the spatiotemporal analysis of gestures using wearable sensors is needed to improve understanding of this link between impairments, functional capacities and task performance in ecological context thanks to behavioral or technological compensations.

## Clinical Perspectives

### Better Understanding Each Individual Patient’s Dexterity

As described above, hand dexterity after stroke is multifactorial since there is no linear causality between the severity of the brain lesion, in particular that of the CST, and the functional independence of the person. The explanation is likely the possibility of compensation at several integrative levels (Levin et al., 2009). Biological compensation is achieved by plasticity and vicariance at the risk of maladaptive phenomena. Functional sensorimotor compensation is achieved by tuning spatiotemporal motor patterns thanks to kinematic redundancy across the different segments of the body (trunk, upper-limb, hand and fingers) and adaptation of grip forces (adjusted safety margins) (per the training and rehabilitation cycle indicated in Figure 2). As a consequence, patients with moderate recovery from stroke may execute motor plans roughly similar to those of healthy controls (i.e. with similar end-point trajectory) despite the impairment of the fine sensorimotor control. Compensation during naturalistic activity is achieved through the planning and execution of alternative motor strategies according to the context and the environment. The most prominent strategy is the exclusive use of the less affected limb; but patients also frequently use specific reaching and grasping strategies during unimanual or bimanual activities, with or without the assistance of technical devices. Compensation at any given level may have complex reciprocal influences upon any other level, leading to the emergence of a wide variety of upper limb motor behaviors post stroke.

Various clinical evaluation tools are available for testing (Santisteban et al., 2016; Villepinte et al., 2020). However, scores obtained through clinical tests of upper limb function do not precisely distinguish the pathophysiological link between the consequences of brain lesions and the multi-level compensatory mechanisms. Based upon the review presented here, we reiterate previous calls for the development of kinetic and kinematic methods in the clinical settings which may complement clinical scores (Nowak, 2008; Winstein and Varghese, 2018; Kwakkel et al., 2019). At this point though, quantitative analyses of upper limb motor behavior in clinical settings are generally based on highly constrained movement tasks, adapted from those used in experiments on healthy individuals. We advocate further studies to quantify more naturalistic tasks similar to daily life tasks, including manipulation of objects and tools as well as pertinent bimanual activities. The short-term objective would be to distinguish movement characteristics directly consequent to brain lesion from those which emerge through compensation so as to envisage more personalized approaches to patient rehabilitation.

### Technology for Assessment and Rehabilitation at Home

In effect, quantitative movement analysis of prototypical gestures in clinical settings may be effective for evaluating functional capacity of the upper limb but provide comparatively less information regarding dexterous use of the hand as it pertains to participation in ecological situations. Activity and independence might conversely be investigated in the patient’s natural environment (home or supported care) where daily life gestures would be facilitated (or impeded) by the physical organization and supports available in those surrounds. The increasing sophistication of technological aids (sensors, wearable devices, and home automation) available for use in the home represent an important means for expanding knowledge on patient movements and strategies and for prolonged rehabilitation in the home (Dobkin, 2017; Maceira-Elvira et al., 2019; Jones et al., 2020). While the majority of consumer wearables are focused are focused on tracking some vital signs (heart rhythms and body temperature) or global activity level, extensive research is currently being conducted on the development of wearable systems relying on different technologies (accelerometers, Inertial Measurement Units-IMUs, wearable robotics or EMG sensors) for monitoring and providing feedback on upper body posture and upper-limbs movements (Wang et al., 2017). However, instrumented clothing designed to monitor activity in daily life is not yet a reality (Maceira-Elvira et al., 2019). Effectively measuring the highly precise movements of the hand using wearables also remains a complex challenge (Lin et al., 2017), with instrumented gloves being complex to install and calibrate (particularly on paretic hands of stroke patients). The availability of relatively inexpensive motion capture systems relying on depth or stereoscopic cameras and without the necessity of worn markers (e.g., Kinect^®^ and Leap Motion^®^) (Guzsvinecz et al., 2019) may provide new avenues for kinematic analysis of hand function. As shown earlier, the characterization of interaction forces by instrumented objects may be a key to the comprehensive examination of manual dexterity and its recovery. In addition, they may be used as “smart toys” for rehabilitation exercises (Hussain et al., 2015; Borghese et al., 2019). Wearable EMG sensors could also estimate underlying muscular activations in post stroke patients (Mendez et al., 2017). Wearable solutions as reviewed above can be used to provide feedback to the patient to assist and encourage home based rehabilitation exercises. Some commercial products exist, such as the Armeo^®^Senso by Hocoma, which relies on a set of worn IMUs and a visual interface to guide patients during rehabilitation. Using embedded sensors in smartphones to track upper limb movement could also be a simple, accessible solution to simplify and generalize assessment and monitored home rehabilitation (Ferreira et al., 2014).

Finally, while a growing number of measurement solutions are becoming accessible to assess dexterity, standardized approaches to processing the complex multidimensional datasets which they produce will need to be consolidated (Appelboom et al., 2014). Automated processing and flexible visual analysis tools are essential in order to extract meaningful information which the clinician may use to inform therapeutic interventions.

### Therapeutic Indications

The choice of a rehabilitation intervention is based on an overall clinical evaluation of the individual patient, with complementary analysis provided through brain imaging and functional tests. The distinction between recovery and compensation is nonetheless crucial to the matrix of clinical decision making for rehabilitation. The atypical compensatory motor pattern used by the patient may represent a viable adaptation given their physical capacities with respect to the environmental constraints (Latash and Anson, 1996). However, compensatory patterns can inhibit recovery of normal motor behavior due to learned non-use phenomena (Hidaka et al., 2012), or worse, exacerbate physical deformity (e.g., contractures or orthopedic complications). It is thus important to consider individual movement patterns in order to decide whether the therapeutic intervention should limit compensations and attempt to improve the impairment (true recovery), or to proceed with training compensatory patterns with the objective of improving movement safety, supporting functional independence in ADLs and promoting social participation. These options are not necessarily compatible given that therapies which pursue true motor recovery can be quite demanding, necessitating strong motivation over prolonged periods of time (typically several weeks or months). In contrast, favoring compensation may lead to a more immediate benefit. However, such processes are complex to analyze due to (i) intricate causal chains between the state of brain structures, upper-limb function and activity routines, and; (ii) functional discrepancies between behavior (learning) and neurobiology (plasticity). The aim of this section is to underline several crucial elements which might be considered when planning rehabilitation for chronic stroke patients rather than to review rehabilitation methods.

#### Promoting Recovery by Exercise

Ideally, therapies should induce recovery at the level of the brain via cortical networks, once the extent of the lesion is stabilized following the acute period. Many promising neurobiologically inspired interventions have been proposed (non-invasive brain stimulation and neuro-technologies) but are yet to demonstrate their effectiveness in routine clinical practice (Sandrini and Cohen, 2013; Regenhardt et al., 2020). Regardless, activity-dependent neural plasticity remains the cornerstone of contemporary advances in rehabilitation practice (Nudo et al., 1996). As demonstrated by Nudo et al. (1996), following a lesion to the M1 cortical representation of the hand in monkeys, recovery of dexterous upper limb function occurred only among those monkeys who completed functional exercise by grasping food in feeding activities. The dimensions of the cortical representation for monkeys with no specific training program (spontaneous recovery) were found to diminish. This contraction of cortical maps supports the behavioral concept of learned non-use. Structural plasticity (synaptogenesis, axonal growth and branching) in regions proximal to the lesion (premotor area) was later demonstrated in animal studies (Dancause, 2006). Nudo’s observations are accepted as proof that plasticity of the cortical map is the neurobiological basis of true recovery. However, a video analysis of the same monkeys in a complementary article showed that some of them, in fact, used alternative grasping strategies (Friel and Nudo, 1998). In stroke patients, recovery through the subacute period is associated with changes in brain excitability, functional plasticity of cortical maps, and changes in connectivity in both hemispheres [review in Loubinoux et al. (2017)]. But the clinical consequences of these processes still remain unclear since functional neural plasticity does not necessarily lead to behavioral recovery (Buma et al., 2016; Peters et al., 2017). Structural plasticity probably contributes to improvements during long term rehabilitation but the relationships between the physical intervention and structural plasticity, and between structural plasticity and clinical outcome are still unclear [review in Sampaio-Baptista et al. (2018)].

In healthy subjects, the improvement of performance with repetition during sensory-motor learning is composed of two processes, occurring at different time scales: adaptation and skill learning (Kitago and Krakauer, 2013). Adaptation corresponds to the tuning of sensory-motor parameters to the actual situation after a relatively small number of repetitions; it relies on functional excitability changes and plasticity in the brain and cerebellum sensory-motor networks. Skill learning requires long-term practice, typically in a professional, sporting or artistic context, and can lead to structural changes in the central nervous system, as demonstrated in healthy subjects (Sampaio-Baptista et al., 2018). Contemporary rehabilitation methods inspired by motor learning paradigms are mostly based on the active repetition of meaningful movements, in contrast to classical neuro-developmental methods. The practice should be task specific, goal oriented, and motivating, with intense well-structured practice and provision of adequate sensory-motor feedback (Krakauer, 2006; Maier et al., 2019). Sensory-motor learning is often assisted by technology (e.g., virtual reality, robotics, and adapted video games) to provide more precise and standardized exercises and increased motivation thanks to engaging game design and user experience. However, it is still unclear if patients can truly recover after the acute, 3 month period of spontaneous recovery. Recent Cochrane meta-analyses showed that robotics and electromechanical devices could improve ADL, arm function and strength (Mehrholz et al., 2018) while the benefit of virtual reality was less convincing but significant when used in addition to standard care (Laver et al., 2017). Both studies underline the difficulties involved in evaluating the efficiency of these methods, as considerable variation is observed across interventions from one trial to another, and between the characteristics of the control intervention (in particular, “usual care,” which is still a “black box,” or matched intensity exercises).

#### Can the Patient Truly Recover Pre-morbid Motor Function?

The possibility of recovery at the impairment level is particularly debated. Negative findings could be due to a dose-effect (Winstein et al., 2019). The cumulated duration of training during usual trials is relatively low [18–36 h according to Ward et al. (2019)] and increasing the dosage up to 60 h improved the MAL but not function (WMFT) (Winstein et al., 2019). A recent study used a particularly intense schedule (300 h in 60 sessions with 5 h/day training) to compare three rehabilitation methods (robotics, functional electrical stimulation, and motor learning). The authors observed some significant and clinically relevant improvement at both impairment (Fugl-Meyer) and activity level, irrespective of the method used. Accordingly, a recent, non-controlled, retrospective study suggested that particularly intensive and prolonged therapies in stroke patients could lead to some improvement in the Fugl-Meyer score (Ward et al., 2019). Other studies suggest that training based on individual finger movements could induce some improvements that generalized to patient performance on Fugl-Meyer, hand function (ARAT) and activity (MAL) testing (Mawase et al., 2020). Similar approaches using highly specific hand training tasks (aiming, tapping, turning…) have also been found to improve manual dexterity when evaluated using the Fugl-Meyer test and hand activity test (TEMPA, Test d’Evaluation des Membres Supérieurs de Personnes âgées) (Platz et al., 2009; Platz and Lotze, 2018).

#### What Is the Behavioral Effect of Constraint Induced Movement Therapy?

The most studied method to limit compensation is Constraint Induced Movement Therapy (CIMT) and its modified derivatives. The principle, proposed by Taub, is to impede the use of the less affected limb while soliciting the most affected limb with an intensive training program (Taub et al., 1999). This method is indicated only to patients who have already attained a certain functional threshold (clinically determined by taking into consideration factors such as partial recovery of wrist and finger extension) but has nonetheless provided a source of much hope (Sirtori et al., 2009). However, a recent meta-analysis of CIMT in chronic stroke patients reported “limited improvements in motor impairment and motor function, but that these benefits did not convincingly reduce disability” (Corbetta et al., 2015). In particular, a kinematic study showed no improvement in coordination during a 2D pointing task (Kitago et al., 2013). These inconsistencies could be due to individual differences, with some patients improving enough to use their limb, while others regressing following conclusion of the training period (Han et al., 2008). Improvement in daily life activity and self-reported arm use after constraint induced therapy could be attributed to the learning of new behavioral compensations, possibly involving both limbs and not to the recovery of the impairment [review in Kwakkel et al. (2015)].

#### Movement Quality Is Important

When the use of the less affected limb is blocked during CIMT, people can still use compensatory motor patterns based on body redundancy, in particular the participation of the trunk. This is the equally true of conventional rehabilitation exercises, when the success of the task is only based on the displacement of the end-point. In contrast to classical rehabilitation methods such as Bobath (Levin and Panturin, 2011), these methods based on goal success seldom consider the quality of movement performance. As described in Section “Kinematics of Reach to Grasp Movements,” stroke patients may preferentially involve the trunk instead of exploiting shoulder and elbow rotation to displace the hand toward the target. The involvement of the trunk may be detrimental by inducing non-use phenomena of the upper-limb. Indeed, the limitation of trunk compensation by a physical restraint can improve shoulder-elbow coordination (Michaelsen et al., 2001). Limiting trunk compensation can thus “unmask latent potential recovery of upper extremity movement” (Wee et al., 2014). Training better movement coordination increases the efficiency of training as shown by the initial study (Michaelsen et al., 2006) and a meta-analysis which showed a moderate effect on the impairment and on the kinematics (Wee et al., 2014). These studies show that the quality of movement coordination during training is important and should be closely controlled either by trunk restraint, by Knowledge of Performance (KP) feedback given by the therapist (Cirstea and Levin, 2007) or by specific technology assisted KP, for example auditory feedback (Chen et al., 2016).

#### Task Related Skill Training

Some rehabilitation methods based on skill training aim at improving daily life activity, rather than reducing impairment through repeated movements (Winstein et al., 2016b). Rehabilitative task-oriented training can induce dosage-dependent improvements in reported motor activity (MAL) (Winstein et al., 2019). However, the effect of skill training is controversial since the interventions are difficult to systematize, and meta-analyses have shown only modest effects (French et al., 2010; Timmermans et al., 2010).

One difficulty is that very few studies have addressed the training of bimanual tasks, which are essential for daily life. A pilot study suggested that intensive training of the ipsilesional, less-affected, limb could improve its dexterity (Jebsen test) and could generalize to functional independence (Sainburg et al., 2016). Several studies developed symmetrical bilateral arm training with the perspective of assisting the paretic limb by the less affected limb thanks to interlimb coupling (Whitall et al., 2011), however, a meta-analysis did not show any clear neural or behavioral effects (Choo et al., 2015). Surprisingly, there are very few studies of bimanual rehabilitation methods with more functional, asymmetric manipulative tasks in hemiparetic patients despite their relevance for daily life activity. A better understanding of the use of both hands during manipulative actions is needed, particularly the effect of laterality and handedness.

Individual behavior during ADL according to the mantra “use it and improve it or lose it” is probably a key to better understanding of why some patients above a certain functional threshold continue to improve during follow-up while other regress (Hidaka et al., 2012).

## Conclusion

Interpreting functional movements such as prehension or manipulation in pathological populations implies an inherent dilemma since they represent both the consequences of the lesion in association with the measures taken to overcome those limits. Movements are not pathological in and of themself; but simply the vehicle for intended actions. Broadly speaking, patients with mild to moderate hemiparesis retain the global spatial organization of prehensile movement in the context of goal directed reaching, multi-finger actions during grasp and grip-load force coordination. Nonetheless, the neurological effects of the cerebrovascular accident bring about various irregularities in upper limb kinematics and dexterous control of the hand. These different features may be related to different underlying processes. Certain components of the movement may be directly related to the CST lesion, in particular alteration of the more refined individual finger control and precision grip. While other movement irregularities would be associated with mechanisms implicated in the reorganization of the nervous system (hyperexcitability, plasticity, and vicariance) and of body coordination (e.g., use of body redundancy and setting of force level). In addition to this, alternate strategies of voluntary movement emerge as the patient exploits the abundant motor solutions offered across the brain-body-environment system, leading to behavioral changes in upper limb activities.

While clinical observations attest to the importance of multidisciplinary physical rehabilitation, the precise ingredients required to stimulate and optimize the recovery of upper limb function and manual dexterity post stroke remain elusive. Available international guidelines enumerate recommended rehabilitation methods (e.g., Winstein et al., 2016a). However, as recently pointed out by Bernhardt et al. (2019) “Clinical trials and observational studies have so far failed to distinguish behavioral restitution from behavioral substitution, leaving the association between quality of movement and recovery of upper limb capacity underexplored.” Many studies lack a precise description of the intervention and of the resulting motor behavior. The principles of motor learning involved should be completely described (Maier et al., 2019) with precise dosage, repetition, duration and intensity (Timmermans et al., 2010). The objective of the methods in terms or recovery/compensation at the different levels (brain networks, body function, activity, and participation) should be clarified to facilitate the interpretation of their pathophysiological effects. To this end, standardized instrumented evaluations should be recommended to measure the quality of movements and to distinguish recovery and compensation (Kwakkel et al., 2019; Alouche et al., 2020).

Meta-analyses generally show disappointing negative results but often underline the great heterogeneity of the interventions under review, for example virtual reality or robotics. Randomized controlled trials are the golden standard of evidence-based medicine. However, it is impossible to evaluate separately all the “rehabilitation ingredients” constituting a given intervention without a combinatorial explosion. In addition, it is difficult to account for individual variability beyond simple stratification. A current reflection examines alternative possibilities of “practice-based evidence research” intermediate between randomized controlled trials and retrospective clinical observations (Horn et al., 2012). Future trials could also be inspired by statistical protocols used for precision individualized care (Janiaud et al., 2019).

Many technologies, wearable or not (neurotechnologies, virtual reality, robotics, games, telerehabilitation etc.) are being developed in order to increase the intensity and duration of therapeutic activities in the home environment (Dobkin, 2017; Maceira-Elvira et al., 2019; Jones et al., 2020). Whilst the description of technology assisted rehabilitation methods is beyond the scope of the present article, it is worthy to note that continued fitness exercises as part of home rehabilitation programs are crucial for maintaining and improving motor functioning of both upper limbs in the long term (Ward et al., 2019). Moreover, if a person has reached a sufficient functional level of dexterity to use his/her limb during daily life activities, he/she will have greater opportunities to improve further (Han et al., 2008). This is especially true if the most affected limb is regularly engaged in bimanual actions. There is probably a virtuous circle between prolonged home or community-based rehabilitation and activities of daily life.

## Author Contributions

AR-B wrote the first draft of the manuscript. RP and NJ wrote sections of the manuscript. All authors contributed to manuscript revision, read, and approved the submitted version.

## Conflict of Interest

The authors declare that the research was conducted in the absence of any commercial or financial relationships that could be construed as a potential conflict of interest.
